# School structure, bullying by teachers, moral disengagement, and students’ aggression: A mediation model

**DOI:** 10.3389/fpsyg.2022.883750

**Published:** 2022-09-05

**Authors:** Valeria Ivaniushina, Daniel Alexandrov

**Affiliations:** Department of Sociology, HSE University, Saint Petersburg, Russia

**Keywords:** school climate, school discipline, moral disengagement, bullying by teachers, peer aggression, victimization

## Abstract

**Aim:**

Understanding interrelations between the factors predicting students’ aggressive behavior is a priority for bullying-prevention programs. Our study explores two possible mechanisms linking school disciplinary structure and students’ aggression. We test students’ moral disengagement and bullying by teachers as mediational pathways from school authoritative discipline to students’ aggressive behavior.

**Methods:**

We used a regionally representative sample of 213 schools that participated in a school climate survey in Kaluga Oblast (a federal subject of Russia) in 2019. The analytical sample contained the anonymous responses of 16,809 students from grades 6–9 (12–15 years old); 51% of the respondents were girls. The analytical procedure consisted of structural equation modeling (SEM), which was implemented in Mplus 8.7.

**Results:**

The mediation model fit the data well, suggesting that the clarity and fairness of school rules negatively predicted peer aggression, while student moral disengagement and bullying by teachers independently and partially mediated this association.

**Conclusion:**

We confirm that authoritative school climate, characterized by a clear and fair disciplinary structure, is associated with a decrease in bullying. Novel result is the evidence for mediating mechanisms and the influence of teachers’ aggression on students’ behavior. Prevention programs designed to increase the fairness and consistency of school rules, eliminate bullying and humiliation from teachers, and decrease students’ moral disengagement may reduce violence and victimization at school.

## Introduction

School aggression is a serious problem found in various cultures and societies all over the world, as evidenced by representative cross-national studies (Chester et al., 2015). While a decreasing trend in bullying has been observed (Cosma et al., 2020), the rates remain troubling. On average, 10–15% of schoolchildren experience bullying during a school year; some studies have reported even higher rates of victimization (Modecki et al., 2014; Cosma et al., 2020). Understanding the factors and the pathways leading to school aggression is necessary for designing effective anti-bullying interventions, and researchers, practitioners, and policy-makers are constantly seeking evidence-based practices to decrease school aggression and promote students’ physical and emotional safety in schools (Meyer-Adams and Conner, 2008; Hong and Espelage, 2012; Cornell et al., 2016).

According to Bronfenbrenner’s (1994), the risk factors associated with aggression act on different levels. These factors include relations with peers, parents, and teachers, as well as cultural norms and school structure (Hong and Espelage, 2012). Disorganized environment, whether it is a dysfunctional family, disordered school, or violent neighborhood, has been shown to affect children’s development and influence their behavior (Allen et al., 1998; Wang and Maguire-Jack, 2018). Schools is second to the families as most important institution providing immediate environment in which children’s social and behavioral development takes place.

Researchers have shown that school environment as a whole and its specific parameters exert considerable influence on adolescents’ aggressive attitudes and behavior (Bradshaw et al., 2009; Saarento et al., 2013). Much effort is put into bullying prevention using whole school approach thus confirming the role of collective school climate in preventing students’ aggression (Richard et al., 2012). In particular, teachers’ attitudes toward bullying have been shown to influence students’ behavior (De Luca et al., 2019).

The idea of observational learning stating that children learn from observing and imitating the behavioral models provided by adults and peers is the cornerstone of widely accepted socio-cognitive theory by Albert Bandura (2001). Social cognitive theory emphasized the importance of cognitive processes, moral cognition in particular, as a regulator of aggressive behavior. Researchers developing Bandura’s ideas posit that moral disengagement shared between group members can be a part of school culture, and collective moral disengagement is a contextual factor affecting individual behavior (Thornberg et al., 2021).

While there is extensive literature on peer influence and the influence of parents, including parental physical and psychological aggression (Kuppens et al., 2009; Farrell et al., 2011; Faris and Ennett, 2012), there is considerably less literature on the effects of teachers’ aggression on the aggressive behavior of students. A few studies showed that aggressive classroom management is relatively wide-spread (Lewis et al., 2005; Romi et al., 2011), but little is known whether aggressive style in classroom management leads to aggressive behavior of students.

The present study aims to explore and evaluate the relationship between school climate, bullying by teachers, moral disengagement, and students’ aggression by means of structural equation modeling (SEM) in order to analyze the mediating mechanisms underlying this association.

## School disciplinary structure and aggression

The prevalence of bullying varies dramatically across schools. Attempts to explain the differences in bullying *via* structural characteristics, such as school size, location, and socioeconomic status, have yielded inconsistent results (Whitney and Smith, 1993; Ma, 2002; Bradshaw et al., 2009; Saarento et al., 2013). One possible reason for the variances in bullying rates is the differences in school climate and school discipline.

Positive school climate has been found to be inversely related to aggression and violence (Meyer-Adams and Conner, 2008; Guerra et al., 2011). The indicators of school climate that have been associated with school violence are school connectedness (Acosta et al., 2019; Markkanen et al., 2021), interpersonal relationships (Acosta et al., 2019; Varela et al., 2019; Markkanen et al., 2021), and disciplinary structure (Gregory et al., 2010; Cornell and Huang, 2016).

Authoritative school climate is based on demanding and responsive behavior of teachers and administrators (Gregory et al., 2010), and it is conceptualized as having two components: disciplinary structure, formed by clear and relatively strict rules, and student support, formed by responsive and effective teacher-student relations (Cornell and Huang, 2016). In a series of articles Dewey Cornell, Ann Gregory and their coauthors have shown that strict and fair discipline and the consistency and clarity of school rules are associated with lower prevalence of multiple forms of negative behavior—from bullying to carrying weapon to school to sexual peer harassment (Gregory et al., 2010; Cornell, 2015; Cornell and Huang, 2016; Crowley et al., 2019).

Several authors have emphasized the need for better understanding of the relationship between school discipline and adolescent violence, pointing out that school discipline may indirectly impact student aggression *via* intermediate mechanisms (Benbenishty et al., 2016; Acosta et al., 2019). Understanding these mediating mechanisms is a key precondition for developing effective anti-bullying interventions.

## Moral agency and peer bullying

Bullying is considered to be unfair and immoral behavior. Most people have sufficient moral knowledge to understand that bullying is wrong. Nevertheless, many children and adolescents commit acts of moral transgression that affect other people’s wellbeing (Thornberg and Jungert, 2014).

To explain the discrepancy between moral reasoning and immoral conduct, Bandura et al. (1996) proposed the concept of moral disengagement. It is a cognitive mechanism that inhibits the connection between moral standards and immoral behavior, thus allowing individuals to rationalize and justify antisocial behaviors, such as aggression and bullying. Researchers have described several disengagement practices, including moral justification through euphemistic labeling; minimization of, or disregard for, detrimental effects; displacement or diffusion of responsibility; and victim blaming (Bandura et al., 1996; Bandura, 2001).

Moral disengagement has been examined in the context of school bullying. Several studies have demonstrated associations between high levels of moral disengagement and student aggression (Bandura et al., 1996; Thornberg et al., 2017; Killer et al., 2019). Meanwhile, students demonstrating prosocial and defending behavior tend to score low on moral disengagement scales (Thornberg and Jungert, 2014; Romera et al., 2019).

Moral disengagement can be viewed on both individual level and on the group level. The concept of collective moral disengagement introduced by Bandura was used in bullying studies to describe shared group beliefs that morally justify negative actions (Gini et al., 2015); and in a recent study lower collective moral disengagement was shown to be associated with lower bullying rates (Thornberg et al., 2021). These findings emphasize the importance of investigating moral disengagement in different contexts.

## Bullying by teachers

Teachers play a key role in every aspect of school life, and the relations between students and teachers affect the students’ academic, social, and behavioral outcomes. Farmer et al. (2011) metaphorically described the teacher as an “invisible hand” that influences school social dynamics: first, the teacher can directly observe and manage the peer interactions in the classroom; second, trustful and supportive relations with the teacher are beneficial for peer relations; and third, competent leadership encourages positive classroom behavior. Teachers establish the context for the social development of students and act as role models. Classes in which teachers are socially and emotionally competent are characterized by low levels of conflict and aggression (Jennings and Greenberg, 2009; Farmer et al., 2011).

Various studies have demonstrated that the quality of teacher–student relationships is predictive of peer victimization (Cornell and Huang, 2016; Thornberg et al., 2017). However, research on teachers’ roles in school bullying has been primarily focused on the respectful, trustful, and supportive relations between teachers and students and on teachers who set norms for positive behavior. Research on teachers’ aggressive behavior is scarce, but the existing work shows that it is not rare in developed countries (see, for example, Lewis et al., 2005), and it was shown that aggressive classroom management influence students’ behavior in multiple ways (Romi et al., 2011).

While sufficiently less prevalent than bullying by students, bullying by teachers is not less harmful. Students who are bullied by teachers develop negative views of school and learning, exhibit lower school engagement, and have poorer academic performances (Delfabbro et al., 2006). Specifically in terms of influence on bullying German authors demonstrated that bullying by teachers and students’ aggressive behavior are positively related (Baier and Kunkel, 2016).

These studies have demonstrated that bullying by teachers is a serious problem in modern schools, and, indeed, there are considerable differences among schools regarding the levels of maltreatment by school staff (Khoury-Kassabri, 2006). Owing to the large power imbalance between students and teachers, students have little or no ability to defend themselves (Whitted and Dupper, 2008). Researchers have noted that bullying by teachers or school staff is a delicate topic to broach (Zerillo and Osterman, 2011; Datta et al., 2017), and school-based efforts to address the problem are necessary.

## The present study

The present study is based on the large data set collected in Russian secondary schools. According to the results of several representative surveys, the prevalence of bullying in Russian secondary schools is 13–16% (Inchley et al., 2020; Avanesian et al., 2021; Ivaniushina et al., 2021). The Health Behavior in School-Aged Children (HBSC), a WHO cross-national study, reports that in 2018 rates of bullying in European countries varied from 3 to 29%, with all-country average 10% (Inchley et al., 2020). Thus, in terms of bullying prevalence, Russia is a rather typical country. There is no quantitative research on teachers’ aggression or school structure in Russia, but there is no reason to think that Russia differs much in this respect from other countries. We assume that the relation between teachers’ aggression and students’ behavior is not country-specific.

In this study we aim to clarify the mechanism(s) underlying the relation between school disciplinary structure and the prevalence of peer aggression in schools. Using social cognitive theory as guiding theoretical approach, we examine whether moral disengagement and bullying by teachers mediate the link between authoritative school rules and peer aggression. Following Bandura’s reasoning on the relation between the lack of moral standards, diffusion of responsibility and moral justification of detrimental conduct (Bandura et al., 1996) we assume that the lack of school disciplinary structure is conducive to both moral disengagement among students and aggressive classroom management techniques by teachers, which in their turn influence students’ behavior allowing more aggression to be manifested in everyday life school interactions. Based on this theoretical consideration, the following hypotheses are formulated:

Hypothesis 1. The prevalence of bullying is negatively related to perceived school disciplinary structure. This hypothesis is based on school climate studies conducted on American schools (Cornell, 2015; Cornell and Huang, 2016); however, this relationship has not been investigated in other cultural contexts. Therefore, this link must be established before we proceed to examine its possible mechanisms.

Hypothesis 2. Bullying by teachers is positively related to the prevalence of peer aggression (H2b) and is negatively related to school disciplinary structure (H2a), thus mediating the relation between disciplinary structure and peer aggression. There is a lack of works that examined the relation between bullying by teachers and peer aggression. One such study conducted on a representative sample of German schools demonstrated that bullying by teachers and students’ aggressive behavior are positively related (Baier and Kunkel, 2016). However, to the best of our knowledge, the relation between school disciplinary structure and bullying by teachers has not yet been studied.

Hypothesis 3. Moral disengagement mediates the relation between disciplinary structure and peer bullying: it is negatively related to school disciplinary structure (H3a) and positively related to the prevalence of peer bullying (H3b). While the relation between moral disengagement and students’ aggressive behavior is well documented (Gini et al., 2015; Thornberg et al., 2021), there is almost no literature addressing the connection between school disciplinary structure and the moral disengagement of students. One recent study did find that the clarity of rules predicts empathy and victimization, but not moral disengagement (Montero-Carretero et al., 2021).

**FIGURE 1:**
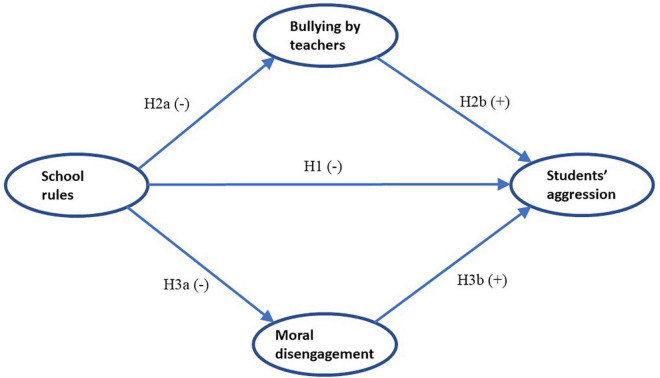
Hypothesized model.

The hypothesized model is shown in Figure 1.

## Materials and methods

### Participants and procedure

The present study is a part of a larger project on school climate that was conducted in the Kaluga region of Russia from 2016 to 2019 (Alexandrov et al., 2018). All schools in the region enrolling over 100 students participated in this study (213 schools in total). The survey was administered once a year to all students in grades 6–9 (12–15 years old) during this time period. For the current analysis, we used the data from 2019. The initial sample consisted of 18,433 students.

This sample is representative of the Russian secondary school system, as the data came from different types of schools situated in cities, towns, and rural areas. Girls comprised 51% of the sample, and students from grades 6–9 were represented in equal proportions (see Table 1). The questionnaire was internet-based, and the students completed the questionnaires online, in computer classes at school; the average time for completion was 22 min.

**TABLE 1:** Students’ characteristics (*N* = 16,809).

Characteristic	%
**Gender**	
Girls	51
Boys	49
**Grade**	
6th	24.5
7th	25.3
8th	25.1
9th	25.1

The use of self-administered questionnaires in schools is prone to eliciting inattentive or careless responses (Fan et al., 2006). Studies have shown that up to 20% of high school students may provide invalid responses due to various reasons (Meade and Craig, 2012; Jia et al., 2018). To ensure that invalid responses do not compromise study findings, several validity screening procedures have been developed (Curran, 2016).

To check the validity of our sample, we used two methods: à screening validity item (“How many of the questions on this survey did you answer truthfully?”) and the assessment of survey completion time. This combination has been recommended as effective in identifying survey data that should be omitted from analyses (Jia et al., 2018). Ultimately, 1,624 students (8.8%) who failed the validity check or had response times that were impossibly rapid were eliminated, resulting in an analytical sample of 16,809 students.

### Measures

School disciplinary structure was measured with three items from the Disciplinary Structure Scale (Cornell, 2015): “The school rules are fair,” “The punishment for breaking school rules is the same for all students,” and “Teachers at our school make it clear that bullying is unacceptable.” The items had four possible answers ranging from “Strongly disagree” to “Strongly agree.” The omega coefficient for this scale was 0.69, with a 95% CI of [0.68, 0.70].

Teachers aggression was measured with two items adapted from the Bullying by Teachers Scale (Cornell, 2015): “How often this school year did you not want to go to school because you were afraid of teachers?,” and “How often this school year have you been reluctant to answer questions in class because you were afraid the teacher would make fun of you?” The items had four possible answers ranging from “Never” to “Very often.” The omega coefficient for this scale was 0.70, with a 95% CI of [0.69, 0.71].

Peer aggression was measured with two items from the Prevalence of Bullying and Teasing Scale (Cornell, 2015): “This school year, how often did your classmates shove and push weaker students?,” and “This school year, how often did your classmates mock other students and talk nasty about them?” The items had four possible answers ranging from “Never” to “Very often.” The omega coefficient for this scale was 0.73, with a 95% CI of [0.72, 0.74].

Moral disengagement was measured with three items from the Moral Disengagement Scale (Hymel et al., 2005): “Sometimes it’s normal that someone is bullied at school,” “It’s normal for teenagers to mock and taunt each other,” and “It is okay to bully someone you don’t like.” The items had four possible answers ranging from “Strongly disagree” to “Strongly agree.” According to Bandura’s classification of moral disengagement mechanisms, these items signify cognitive restructuring or moral justification: when an individual is convinced that all their peers engage in certain actions, this behavior becomes normalized and no longer self-sanctioned. The omega coefficient for this scale was 0.68, with a 95% CI of [0.67, 0.69].

While initial scale, introduced by Albert Bandura, had 32 items to measure 4 loci and 8 mechanisms of moral disengagement theory, it actually formed a single factor structure and was used as a summed-up composite index. In the work by S. Hymel and coauthors the results of factor analysis of 13-item scale “failed to distinguish the four conceptual categories of moral disengagement strategies” (Hymel et al., 2005, p. 5). Thus we feel justified to use short version created by a panel of experts selected items, which in their opinion reflected moral self-justification and were most relevant for Russian schoolchildren.

### Statistical analysis

All analyses were performed using Mplus software, version 8.7 (Muthén and Muthén, 1998). As all the items measuring latent constructs were measured on an ordinal scale, we used DWLS (diagonally weighted least squares) models with robust standard errors. The models were evaluated using the Satorra–Bentler scaled chi-square test and a series of approximate fit indices (CFI, TLI, RMSEA, and SRMR). All models were adjusted for the complex nature of the sample, with students nested within schools, and standard error computations were carried out using a sandwich estimator.

First, we estimated a measurement model with four constructs (school disciplinary structure, peer aggression, bullying by teachers, and moral disengagement) using confirmatory factor analysis.

To test Hypothesis 1, we estimated a structural equation model relating the prevalence of aggressive behavior with school disciplinary structure.

To test mediation (Hypotheses 2 and 3), a full structural equation model (SEM) with all four constructs was assessed. We estimated the total, direct, and indirect effects of school disciplinary structure on aggressive behavior. The 95% CIs for all indirect effects were calculated using bias-corrected bootstrapping with 1,000 samples (MacKinnon et al., 2004; Preacher and Hayes, 2008).

## Results

The measurement model for the four constructs was estimated using the analytical sample of 16,809 students. Confirmatory factor analysis demonstrated a good fit to the data: chi-square = 132.7 (df = 29, *p* = 0.000); CFI = 0.996; TLI = 0.994; RMSEA = 0.015 (95% CI = [0.012, 0.017]); and SRMR = 0.009. The standardized loadings ranged between 0.69 and 0.86, with a mean loading of 0.76.

A structural model with two latent variables was estimated to examine the unique effect of school rules on student aggression. The model had a good fit: chi-square = 17.3 (df = 4, *p* = 0.002); CFI = 0.999; TLI = 0.998; RMSEA = 0.016 (95% CI = [0.014, 0.021]); and SRMR = 0.005. School disciplinary structure was negatively related with peer aggression: beta = -0.467; SE = 0.014; and *p* = 0.000. Thus, Hypothesis 1 is confirmed.

**FIGURE 2:**
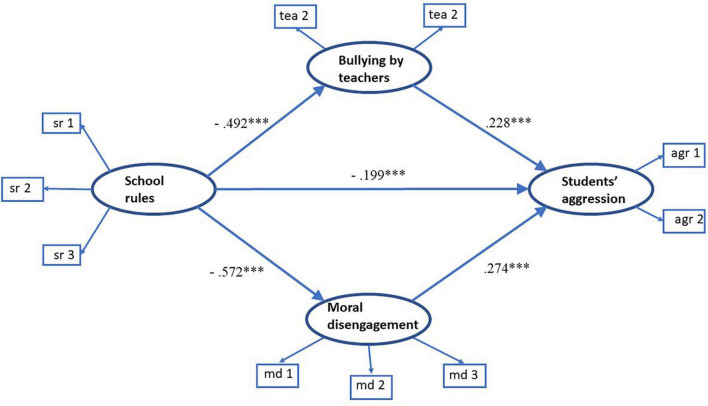
Structural equation model of school disciplinary structure and peer aggression, mediated by bullying by teachers and moral disengagement. The coefficients represent standardized parameter estimates. ^***^p < 0.001.

In the next step, we estimated the full structural model (Figure 2). The model fit the data well: chi-square = 164.8 (df = 30, *p* = 0.000); CFI = 0.995; TLI = 0.993; RMSEA = 0.016 (95% CI = [0.014, 0.019]); and SRMR = 0.011.

All path coefficients in the full model were significant and in line with our hypotheses. School disciplinary structure negatively predicted bullying by teachers (beta = -0.492, SE = 0.015, and *p* = 0.000) and moral disengagement (beta = -0.572, SE = 0.013, and *p* = 0.000). In turn, the prevalence of peer aggression was positively associated with bullying by teachers (beta = 0.24, SE = 0.02, and *p* = 0.000) and moral disengagement (beta = 0.274, SE = 0.018, and *p* = 0.000). This structure of relations between the constructs confirms Hypotheses 2 and 3.

The standardized path coefficients, as well as the direct, indirect, and total coefficients, along with their 95% CIs, are shown in Table 2. The indirect effect *via* bullying by teachers was −0.114, with a 95% CI of [−0.97, −0.132]. The indirect effect *via* moral disengagement was −0.159, with a 95% CI of [−0.138, −0.184]. The total effect of school rules on peer aggression was −0.475, with a 95% CI of [−0.444, −0.510], and the total indirect effect was −0.273, with a 95% CI of [−0.246, −0.304].

**TABLE 2:** Path coefficients (standardized) in the model of school disciplinary structure and peer bullying, mediated by bullying by teachers and moral disengagement.

Path	Estimate	SE	*P*-value	95% CI
School rules Bullying by teachers (H2a)	–0.492	0.015	0.000	
Bullying by teachers Students’ bullying (H2b)	0.228	0.016	0.000	
School rules Moral disengagement (H3a)	–0.572	0.013	0.000	
Moral disengagement Students’ bullying (H3b)	0.274	0.018	0.000	
School rules Students’ bullying	–0.199	0.020	0.000	
Total effect	–0.475	0.017	0.000	−0.444; −0.510
Total indirect effect	–0.273	0.015	0.000	−0.246; −0.304
Specific indirect effect *via* Bullying by teacher	–0.114	0.009	0.000	−0.097; −0.132
Specific indirect effect *via* Moral disengagement	–0.159	0.012	0.000	−0.138; −0.184

Overall, the full structural model accounted for 31.7% of the variability in peer aggression, 26.5% of the variability in bullying by teachers, and 35% of the variability in moral disengagement.

## Discussion

School aggression is common all over the world and has many negative consequences. Understanding the mechanisms underlying bullying is crucial for designing effective prevention and intervention measures. Researchers of bullying urge to identify malleable factors with which to decrease school violence and increase students’ wellbeing (Bradshaw et al., 2009; Hong and Espelage, 2012; Datta et al., 2017; Acosta et al., 2019).

In our study we used a large representative school sample to examine the mechanisms behind the relationship between school disciplinary structure and peer aggression in schoolchildren. All the hypotheses put forth in this study based on theoretical considerations and previous empirical results were confirmed.

Our results suggest that clarity and fairness in school rules is negatively related to peer aggression, while moral disengagement and bullying by teachers independently and partially mediate this association. While the direct association found between school rules and the prevalence of bullying is consistent with the findings in existing research (Cornell and Huang, 2016), to the best of our knowledge, no previous literature has addressed the mediating roles of moral disengagement and bullying by teachers.

Clear school rules and fairly implemented discipline are basic components of an authoritative school climate. Students who are aware of school rules and believe they are fair tend to refrain from violent behavior (Fisher et al., 2018; Crowley et al., 2019). An authoritative school climate provides a healthy balance of supervision, monitoring, and support, helping students handle psychological, emotional, and behavioral challenges. Previous studies relating school disciplinary structure with peer aggression were conducted on US schools (Cornell and Huang, 2016; Fisher et al., 2018; Crowley et al., 2019). Our findings from a large sample of Russian schools corroborate these results across cultural contexts.

Student–teacher relationships comprise another key component of school climate. Perceived respect from teachers and other school staff positively impacts student motivation and academic performance, and trustful relations are related to decreased bullying (Cornell and Huang, 2016; Longobardi et al., 2016; Thornberg et al., 2017). Recent studies have reported that individual teachers’ characteristics, such as self-efficacy, job satisfaction, and specific competence in relation to bullying situations, are negatively associated with the level of bullying and victimization in school (De Luca et al., 2019).

Negative student-teacher relations, such as bullying by teachers, have been explored less often. Thus, we proposed and tested the hypothesis that bullying by teachers mediates the link between school discipline and student aggression; our findings strongly supported this hypothesis. While caring, considerate teachers establish standards of mutual respect in a classroom, teachers who ridicule and belittle their students set example of bullying behavior that might be considered normal by schoolchildren.

Following the social cognitive theory of moral agency (Bandura, 2001), we included in our model moral disengagement as another possible mediating mechanism for the relationship between school disciplinary structure and peer aggression. A positive relationship between moral disengagement and peer aggression has been documented in several studies (Gini et al., 2015; Thornberg et al., 2021). We contribute to the understanding of relations between school rules, aggression, and morality by establishing a link between moral disengagement and school disciplinary structure; when students perceive school rules as clear and fair, they are less inclined to activate mechanisms of moral justification regarding bullying.

We also view our results in the light of social disorganization theory (Sampson and Groves, 1989; Bellair, 2017), which has been already applied to school violence (Bradshaw et al., 2009; Espelage and Swearer, 2009; Plank et al., 2009; Bradshaw and Johnson, 2011). We think that this theory deserves more attention in research on bullying. Thinking along the lines of social disorganization theory provides us with hypothetical mechanism underlying the tested structural model. Both the perceived absence of clear rules in school and the aggressive behavior of teachers are certainly signs of social disorganization. The lack of transparent school disciplinary structure creates ambiguity and alienation of students, which results in moral disengagement, which justifies aggression. When individual moral disengagement becomes prevalent in a group as a collective feature it can become a force in normalizing aggression.

Social disorganization studies of neighborhoods based on application of Albert Bandura’s concepts to collective life have shown the existence of a feedback loop (“vicious circle”) of disorder and social-cognitive perception of potential collective action (Sampson and Groves, 1989). People living in violent environment are getting used to aggression, develop moral disengagement, and lose collective efficacy in dealing with disorder. Because of this justification they either commit more aggression themselves or distance themselves from the acts of aggression (bystanders who are morally disengaged never intervene), thus contributing to the prevalence of aggression in the social environment. By the same logic, collective effort can diminish disorder and build up a virtuous circle of self-sustaining improvement.

Anti-bullying intervention programs in the last decades have been designed and implemented worldwide, and a growing number of prevention programs are available to schools. It has been shown that interventions that focus primarily on student behavior modification have little or no effect (Gaffney et al., 2019; Romera et al., 2019). In their review of research on school bullying, Swearer et al. (2010) attributed the modest effectiveness of several anti-bullying interventions to their lack of a solid theoretical foundation. Research attempting to inform school-based anti-bullying efforts should try to pinpoint malleable factors and mechanisms that not only shape and predict violent behavior, but potentially lead to self-sustained changes in collective student body. Effective interventions should target not only the students involved in bullying but the entire school community, including teachers and school staff (Hong et al., 2018). Accordingly, in this paper, we offer a mediation model that combines school, teacher, and student factors underlying school aggression and hypothesize the social and psychological mechanisms at work in producing effects in question.

### Limitations

This study has certain limitations that must be acknowledged. First, as it utilizes a cross-sectional design, we are unable to make any causal inferences. While the mediation model fits the data well, it can only be concluded that the proposed model does not contradict the data, but it doesn’t prove cause–effect relations. Future research could adopt a longitudinal design to examine causal relations between the constructs.

Second, methodologists have stated that mediation analysis with unmanipulated mediators is prone to bias. Unbiased estimates in non-experimental conditions can only be obtained when the error terms for the mediator and outcome variable are uncorrelated, which is hardly ever the case (Antonakis et al., 2010; Bullock et al., 2010). The only way to obtain unbiased estimates is experimental manipulation; however, there are obvious practical obstacles to experimental mediation analysis, as it is not possible to conduct a controlled experiment involving the variables of interest.

Third, while our analysis was conducted on a large, regionally representative sample including different types of schools, the data were collected in one country. Thus, although our results are generalizable for Russia, to expand external validity to other countries, similar research should be conducted in different cultural contexts. It should be noted, however, that our results corroborate the findings of a previous study on a different student population (Cornell and Huang, 2016).

These limitations aside, the present study provides strong support for exploring the association between school disciplinary structure, moral disengagement, and bullying by teachers, as well as how they are related to rates of peer aggression at school.

### Implications for practice

Understanding the interrelations between school aggression and its underlying factors is important for designing theoretically grounded, effective interventions that promote students’ physical and emotional safety in schools. Our study identified several malleable factors that could be addressed to help decrease bullying. Based on the results, the following intervention strategies are recommended.

First, school administrators should establish clear school rules that ensure a fair and supportive school climate. Students should be aware of the rules and boundaries enforced by the school, such as what behavior is inappropriate, and what the punishment for breaking the rules is. Moreover, punishments should be fair and uniform for everyone. Second, schools must enforce rules that strictly forbid teachers from humiliating or ridiculing students. While hidden psychological or emotional pressure is harder to recognize and handle, it is nonetheless an area in which interventions should be focused. Third, moral education that can counteract skewed moral norms should be put into practice. If students are convinced that certain behavior is normal because everyone engages in it, they must be convinced otherwise. Implementing these recommendations will create safer school environments and help students grow into responsible citizens.

## Data availability statement

The datasets presented in this article are not publicly available because we did not request an explicit consent from the schools and students for the public availability of the data. Requests to access the datasets should be directed to the corresponding author VI (ivaniushina@hse.ru).

## Ethics statement

The study design and questionnaire were reviewed and approved by the HSE Committee on Ethical Assessment of Empirical Research. Written informed consent to participate in the study was obtained of all respondents.

## Author contributions

VI and DA conceived and planned the research. VI performed the statistical analysis and took the lead in writing the manuscript. DA provided critical feedback, helped shape the analysis, and contributed to writing and editing the manuscript. Both authors contributed to the article and approved the submitted version.
